# Applying Machine Learning to Daily-Life Data From the TrackYourTinnitus Mobile Health Crowdsensing Platform to Predict the Mobile Operating System Used With High Accuracy: Longitudinal Observational Study

**DOI:** 10.2196/15547

**Published:** 2020-06-30

**Authors:** Rüdiger Pryss, Winfried Schlee, Burkhard Hoppenstedt, Manfred Reichert, Myra Spiliopoulou, Berthold Langguth, Marius Breitmayer, Thomas Probst

**Affiliations:** 1 Institute of Clinical Epidemiology and Biometry University of Würzburg Würzburg Germany; 2 Department of Psychiatry and Psychotherapy University of Regensburg Regensburg Germany; 3 Institute of Databases and Information Systems Ulm University Ulm Germany; 4 Faculty of Computer Science Otto von Guericke University of Magdeburg Magdeburg Germany; 5 Department for Psychotherapy and Biopsychosocial Health Danube University Krems Krems Austria

**Keywords:** mHealth, crowdsensing, tinnitus, machine learning, mobile operating system differences, ecological momentary assessment, mobile phone

## Abstract

**Background:**

Tinnitus is often described as the phantom perception of a sound and is experienced by 5.1% to 42.7% of the population worldwide, at least once during their lifetime. The symptoms often reduce the patient’s quality of life. The TrackYourTinnitus (TYT) mobile health (mHealth) crowdsensing platform was developed for two operating systems (OS)—Android and iOS—to help patients demystify the daily moment-to-moment variations of their tinnitus symptoms. In all platforms developed for more than one OS, it is important to investigate whether the crowdsensed data predicts the OS that was used in order to understand the degree to which the OS is a confounder that is necessary to consider.

**Objective:**

In this study, we explored whether the mobile OS—Android and iOS—used during user assessments can be predicted by the dynamic daily-life TYT data.

**Methods:**

TYT mainly applies the paradigms ecological momentary assessment (EMA) and mobile crowdsensing to collect dynamic EMA (EMA-D) daily-life data. The dynamic daily-life TYT data that were analyzed included eight questions as part of the EMA-D questionnaire. In this study, 518 TYT users were analyzed, who each completed at least 11 EMA-D questionnaires. Out of these, 221 were iOS users and 297 were Android users. The iOS users completed, in total, 14,708 EMA-D questionnaires; the number of EMA-D questionnaires completed by the Android users was randomly reduced to the same number to properly address the research question of the study. Machine learning methods—a feedforward neural network, a decision tree, a random forest classifier, and a support vector machine—were applied to address the research question.

**Results:**

Machine learning was able to predict the mobile OS used with an accuracy up to 78.94% based on the provided EMA-D questionnaires on the assessment level. In this context, the daily measurements regarding how users concentrate on the actual activity were particularly suitable for the prediction of the mobile OS used.

**Conclusions:**

In the work at hand, two particular aspects have been revealed. First, machine learning can contribute to EMA-D data in the medical context. Second, based on the EMA-D data of TYT, we found that the accuracy in predicting the mobile OS used has several implications. Particularly, in clinical studies using mobile devices, the OS should be assessed as a covariate, as it might be a confounder.

## Introduction

### Background

Mobile health (mHealth) uses smart mobile devices to address various questions in the context of neuroscience, psychology, and medicine. New paradigms, such as ecological momentary assessment (EMA), mobile crowdsourcing, and mobile crowdsensing, as well as mHealth apps, in general, have enabled data collection procedures that surpass many existing methods in gathering valuable medical data by several orders of magnitude [1]. Among others, by using smart mobile devices, data can be gathered in everyday life, on a cost-effective basis, and by adding contextual information sources, such as Twitter or Facebook. As many medical phenomena pose daily variations [2], mHealth technology is predestined to be utilized in this context. Along these trends, many insights have been presented by researchers that show that smart mobile devices can help to establish new data sources in many scenarios [3].

In these data collection scenarios, which are built on the usage of mobile devices and their sensors, one dimension has been less considered so far. It refers to the question of whether the operating system (OS) of the mobile technology being used (eg, iOS or Android) constitutes a valuable information source or confounder for medical data analyses. Or, as another example, is it possible to derive insights if a patient changes the OS during a study when using mHealth apps? As Android and iOS dominate the mobile OS market [4]—with a market share of 99.32% in May 2020 (72.52% Android and 26.80% iOS)—any insights gained based on differences from users regarding these OS types could provide a representative picture for the OS market. Following this, data that were gathered with the TrackYourTinnitus (TYT) mHealth crowdsensing platform for tinnitus patients over 5 years of age are analyzed in this paper. TYT is an mHealth crowdsensing platform that offers iOS and Android apps that can empower patients to learn more about their tinnitus symptoms over time. Tinnitus is the phantom perception of a sound and it is experienced by 5.1% to 42.7% of the population worldwide at least once during their lifetime [5]. The symptoms often reduce the patient’s quality of life. As tinnitus constitutes a chronic condition for which currently no cure or general treatment exists, patients suffering from it crave for new treatment procedures or at least new medical insights. With the idea of EMA, also known as ambulatory assessment or experience sampling, and mobile crowdsensing techniques in mind, TYT was developed by an interdisciplinary team of medical experts, psychologists, and computer scientists.

The development of TYT was motivated by the clinical experience that among many tinnitus patients, tinnitus loudness and tinnitus annoyance vary over time and that patients’ experiences differ in the pattern of these fluctuations. Therefore, the variations are considered to provide new valuable insights in the pathophysiological mechanisms of this chronic condition [6]. To learn more about these fluctuations, TYT applies EMA and mobile crowdsensing to capture them. In EMA, the variable in question (eg, a symptom) is assessed repeatedly in daily life [7]. In mobile crowdsensing, only mobile devices are used for the data collection procedure, while their sensors are used to capture, for example, the GPS position or the external sound level [8]. In contrast, in mobile crowdsourcing, tasks are proposed by a crowdsourcer to a group of individuals, who voluntarily undertake tasks. The undertaking of the task always entails mutual benefit. The user will receive the satisfaction of a given type of need, while the crowdsourcer will obtain and utilize to their advantage what the user has brought to the venture [9]. In contrast to mobile crowdsourcing, mobile crowdsensing relies solely on mobile technology and integrates sensors to collect data. Two recent works that discuss mobile crowdsensing in the context of health care can be found in Kraft et al [1] and Pryss [10]. In TYT, the users fill in a registration questionnaire (ie, static data) and can provide repeated assessments in daily life (ie, dynamic data) afterward [11].

### Objectives

Compared to the existing studies on TYT, this work investigates repeatedly provided EMA datasets from TYT users (ie, dynamic data) and their relation to the mobile OS used. While this study analyzes this dynamic data, a previous study focused on differences between Android and iOS users in the static data given at registration [12]. Contrary to the Android versus iOS comparison of the SmokeFree28 (SF28) smoking cessation app [13], in our study we found no differences in gender, but we did find differences in age for TYT users. However, in Pryss et al [12], we found differences that might be of interest for medical purposes. More specifically, we revealed that Android users reported a significantly longer tinnitus duration than did iOS users, cross-sectionally. Future longitudinal research is necessary to address the question of whether users with longer tinnitus duration prefer Android to iOS or whether users of Android tend to develop longer tinnitus durations than iOS users. In another recent work [14], we investigated differences in Android and iOS users of the TrackYourHearing (TYH) mHealth crowdsensing platform. This platform aims to measure fluctuations in hearing of users with hearing loss. In the TYH study, we found no differences in gender or age, but significant differences were revealed in three questions of the dynamic data that were repeatedly provided. This shows that the dynamic data in combination with the OS are worth being investigated more deeply.

As another current trend, the application of machine learning techniques in different fields is promising. In the medical field, there is a remarkable discrepancy between huge expectations in the potential of machine learning on one side and the current application of this technique on the other [15-19]. Importantly, there is an increasing consensus about its potential in the context of mobile technology [20-23]. However, the application of machine learning to a large group of users of an mHealth crowdsensing platform that gathers EMA datasets is still rare [19,24-27]. As we already found relevant differences between Android and iOS pertaining to the TYT users’ static characteristics at registration [12], this work investigates the following research question: *Is it possible to predict the mobile OS used based on dynamic TYT data with high accuracy using machine learning methods? More specifically, is it possible to predict the mobile OS used based on the repeatedly given daily data provided by the TYT users with high accuracy using machine learning methods?* To the best of our knowledge, thus far, no other work has considered this research question in the given context.

## Methods

### Overview

**Figure 1:**
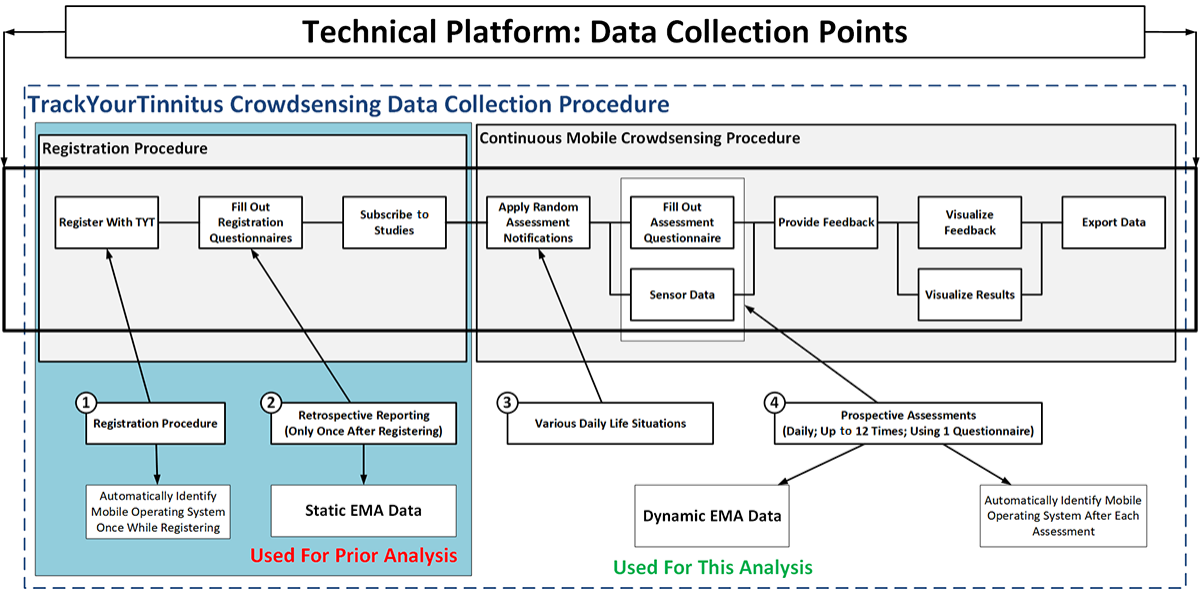
TrackYourTinnitus (TYT) mobile crowdsensing collection procedure. EMA: ecological momentary assessment.

TYT was developed to track the individual tinnitus perception of users in their daily lives [28]. In this context, the procedure shown in Figure 1 is applied to all TYT users. In general, TYT pursues three major goals.

First, dynamic EMA (EMA-D) data shall be collected during the continuous mobile crowdsensing procedure (see Figure 1, box #4). Importantly, a crowdsensing user shall not foresee the times he or she is asked to provide the data (see Figure 1, box #3). This is ensured by asking the crowdsensing users for data in various daily-life situations by the use of smartphone notifications. When a user clicks on such a notification, the tinnitus-tracking questionnaire is presented to a user, consisting of eight EMA-D questions. Table 1 lists the eight questions of the EMA-D questionnaire.

Note that the questionnaire appears visually on both mobile OS types in the same way. For more information on the questionnaire shown in Table 1 and how it appears on the mobile devices, see Pryss et al [11].

Second, the collected data shall enable innovative data analyses, such as juxtaposing the prospectively assessed EMA-D and retrospectively assessed static EMA (EMA-S) at registration (see Figure 1, box #2; [11]). Third, gathered data shall be used to provide feedback to the mobile crowdsensing users [29].

When initially designing the user procedure of TYT, we had not yet considered comparing users based on the mobile OS they used. The initial intention to collect information about the mobile OS used (see Figure 1, box #1) when filling out a questionnaire had been to quickly identify technical issues that could emerge with the large variety of mobile OS versions and mobile devices used. However, it turned out that the information can be also used for innovative analyses. For interested readers, more technical information of the platform can be found in Pryss et al [28,29].

A further note is provided to distinguish between static and dynamic data in the procedure shown in Figure 1. Usually, existing works distinguish between questionnaire, sensor, and behavioral data when utilizing mHealth crowdsensing approaches [30-32]. However, our distinction between static *trait* (ie, EMA-S) or dynamic *state* EMA data (ie, EMA-D) is done less frequently by other works. This is remarkable, as the distinction between trait (ie, static) and state (ie, dynamic) variables is fundamental in clinical and psychological research. As an example, trait data are expected to have a closer association with genetic information as compared to state data, which depend more strongly on environmental factors.

The experimental protocols were approved by the Ethics Committee of the University Clinic of Regensburg, Germany. All methods were carried out in accordance with the relevant guidelines and regulations. The users of the app were informed that their gathered data will be used for scientific analyses; informed consent was given.

**Table 1:** Questions from the dynamic ecological momentary assessment (EMA-D) questionnaire.

Number	Question (Q)	Answer type
Q1	Did you perceive the tinnitus right now?	Yes or no
Q2	How loud is the tinnitus right now?	Slider^a^
Q3	How stressful is the tinnitus right now?	Slider
Q4	How is your mood right now?	Manikins^b^
Q5	How is your arousal right now?	Manikins
Q6	Do you feel stressed right now?	Slider
Q7	How much did you concentrate on the things you are doing right now?	Slider
Q8	Do you feel irritable right now?	Yes or no

^a^Each slider has a different range; the slider for Q2, for example, ranges from *not audible* to *maximal loudness*.

^b^We made use of the Self-Assessment Manikin (SAM) scales, which are a pictorial rating system to obtain self-assessments of experienced emotions on the dimensions affective valence, dominance, and arousal.

### Data Source

The TYT platform includes a website, uses a relational database, and includes an iOS and Android app. The latter are implemented as native apps. Users can register with the platform by using the website or the mobile apps. After that, three registration questionnaires must be completed—EMA-S questionnaires, which can be filled out using the website or the mobile apps—before users can provide the EMA-D data repeatedly in daily life—this is denoted as the EMA-D questionnaire, which can only be filled out using the mobile apps. After completing the registration questionnaires, users must decide whether they want to use the default notification schema for the EMA-D questionnaire. The default setting means users would receive random notifications up to eight times per day. This setting can be changed by a user in many ways. The user can reduce the notification number to a minimum value of three or a maximum value of 12 notifications per day. In addition, a user can select specific days of the week when no notifications shall appear. Finally, a user can switch to the fixed notification mode, in which he or she specifies exact notification points. Note that in this analysis, it is not distinguished which mode has been selected by a user. Finally, if the user clicks on a notification, the EMA-D questionnaire appears. A detailed description can be found in Pryss et al [11]. Finally, note that users can fill out the EMA-D questionnaire in a user-initiated manner as well (ie, without getting a notification to fill out a questionnaire).

Another feature is offered to the TYT users. They can obtain their results of all answered EMA-D questionnaires through the apps or the website. For this purpose, two options are provided: first, they can visualize the results via the website or the mobile apps; or second, they can download a CSV (comma-separated values) file, only via the website, for further personal evaluations.

### Participants

The analysis was conducted in March 2020. At this time, the TYT platform had 4835 registered users. From them, 2584 users completed the EMA-D questionnaire at least once and, in total, 75,278 EMA-D questionnaires were available. To get an impression of how TYT is used worldwide, the country distribution was determined; it shows the number of completed EMA-D questionnaires (ie, all eight items filled in) from 2065 users from the 12 countries with the most completed EMA-D questionnaires out of the 2584 users who completed the questionnaires. This resulted in 67,789 EMA-D questionnaires from 2065 users. The worldwide distribution is shown in Table 2.

The OS distribution of the 2584 users who completed the EMA-D questionnaire at least once is as follows: 40.02% (1034/2584) of the data were provided by iOS users, while 59.98% (1550/2584) were provided by Android users. The OS distribution of all completed EMA-D questionnaires in TYT is as follows: 32.00% (24,089/75,278) of the data were provided by iOS users, while 68.00% (51,189/75,278) were provided by Android users.

**Figure 2:**
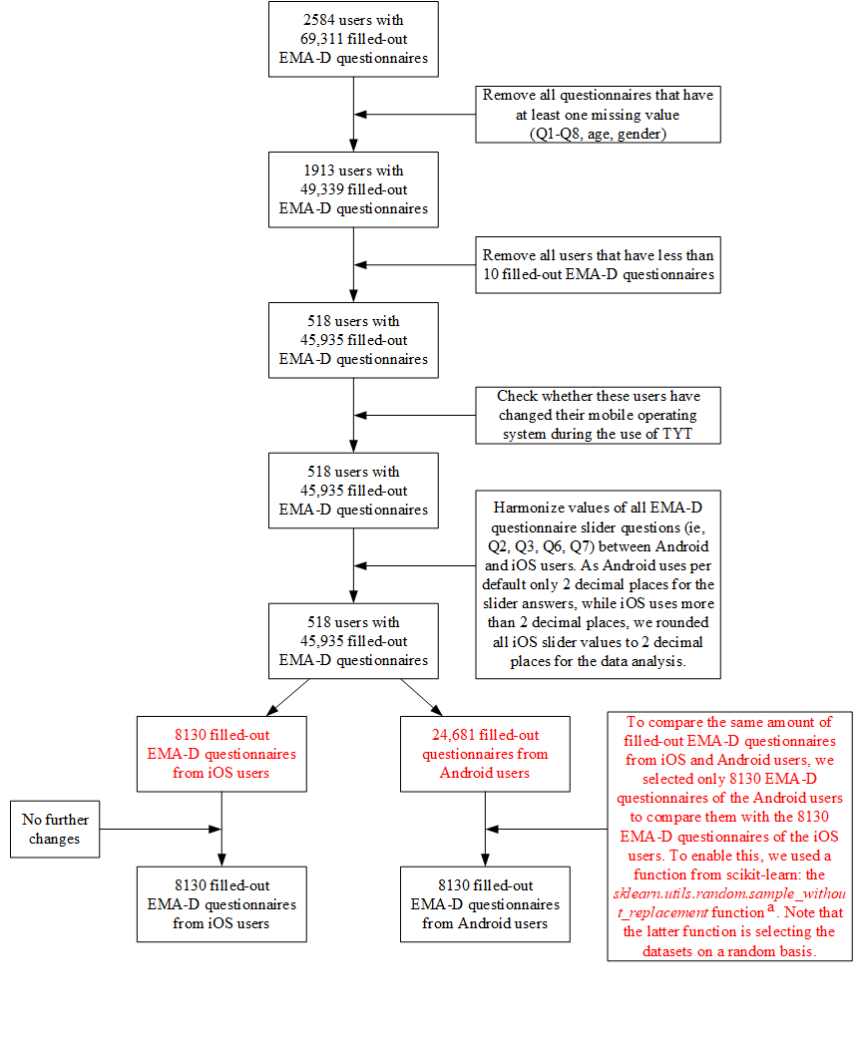
Data preparation steps for the machine learning analysis. ^a^Information about the scikit-learn function can be found on the scikit-learn website [33]. EMA-D: dynamic ecological momentary assessment; Q: question; TYT: TrackYourTinnitus.

The data preparation steps for the machine learning analysis, including use of a scikit-learn function [33] to compare the same number of EMA-D questionnaires from Android and iOS users, can be seen in Figure 2.

For the final study sample of 297 Android users and 221 iOS users, Table 3 shows statistical comparisons between the Android and iOS users in terms of gender, age, and numbers of completed EMA-D questionnaires (chi-square test and *t* tests for independent samples, two-sided). Age was set to *missing* if users provided invalid entries.

**Figure 3:**
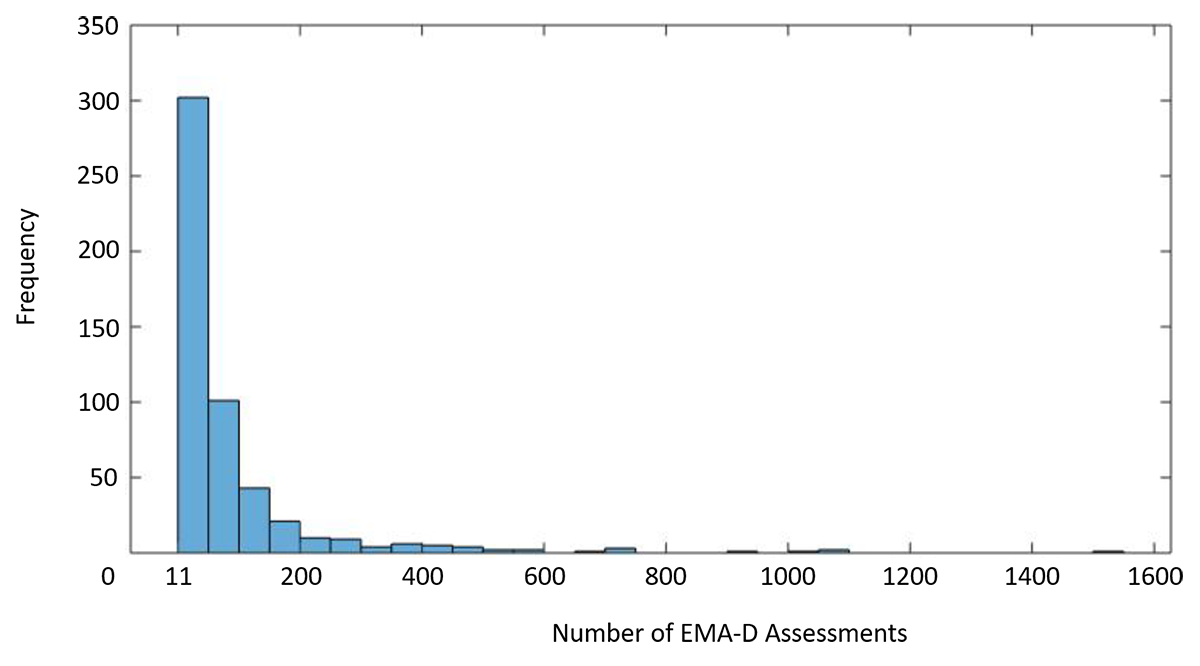
Frequencies of completed questionnaires of the investigated TrackYourTinnitus (TYT) users (n=518). EMA-D: dynamic ecological momentary assessment.

Finally, Figure 3 shows the histogram for the number of completed EMA-D questionnaires for the 518 investigated TYT users (see Figure 2).

**Table 2:** Country distribution of TrackYourTinnitus (TYT) users (n=2065) in ascending order.

Number	Country	Completed dynamic ecological momentary assessment (EMA-D) questionnaires (n=67,789), n (%)
1	Australia	535 (0.79)
2	Belgium	819 (1.21)
3	Italy	1026 (1.51)
4	Russia	1076 (1.59)
5	Austria	1110 (1.64)
6	Norway	1159 (1.71)
7	Canada	2113 (3.12)
8	Great Britain	3202 (4.72)
9	Switzerland	5229 (7.71)
10	Netherlands	6917 (10.20)
11	United States	9117 (13.45)
12	Germany	35,486 (52.35)

**Table 3:** Comparisons between iOS and Android users regarding gender, age, and number of completed dynamic ecological momentary assessment (EMA-D) questionnaires.

Variable	Android	iOS	Chi-square (*df*)	Two-tailed *t* test (*df*)	*P* value
Gender^a^ (male), n (%)	221 (74.4)	147 (66.5)	1.2 (1)	N/A^b^	.27
Age^c^ (years), mean (SD)	49.76 (12.29)	50.57 (13.09)	N/A	–0.71 (497)	.48
Number of completed EMA-D questionnaires^d^ (ie, all eight questions completed), mean (SD)	49.52 (80.32)	66.55 (125.92)	N/A	–1.87 (516)	.06

^a^Sample sizes for gender are n=297 for Android and n=221 for iOS.

^b^N/A: not applicable.

^c^Sample sizes for age are n=295 for Android and n=204 for iOS.

^d^Sample sizes for number of completed EMA-D questionnaires are n=297 for Android and n=221 for iOS.

### Machine Learning Analysis

We applied machine learning approaches with the goal to predict the OS—Android or iOS—of a provided assessment of the EMA-D data. For this purpose, four machine learning approaches were applied to the dataset: a feedforward neural network (FNN), a decision tree (DT), a support vector machine (SVM), and a random forest classifier (RFC). All approaches were chosen because they are appropriate for high-dimensional datasets, which is the case for the given EMA-D questionnaires of the TYT users. This is supported by similar works [34,35].

Concerning the dataset in general, it is noteworthy that the machine learning approaches were applied on the assessment level of the EMA-D questionnaires. This means that assessments from one user can be in both the training and the validation datasets. Performing a separation on assessment level has advantages and disadvantages. As the main disadvantage, it can be argued that if a participant is in both datasets, then there might be a bias. On the other hand, if users of the training phase are separated from the validation phase users, then it must be ensured that the user characteristics between the training and validation phases generate no bias. In an EMA-driven approach, where daily assessments on a random and voluntary basis are the main goal, it is difficult to be able to evaluate a large group of users with similar assessment characteristics. However, in future work, it will be a further goal to also separate the dataset on the user level in a reasonable manner. That individual users play an important role in health care studies is emphasized by the emerging paradigm of N-of-1 studies [36].

Concerning the validation procedure, the following has been accomplished: in a first step, the validation was based on a *10-fold cross-validation* approach [34,35] (ie, for the SVM, the DT, and the RFC). Here, the entire dataset was distributed into 10 equal parts. Nine of these parts were used for the training phase, while the remaining one part was used for the testing phase. The whole procedure was repeated 10 times and the average values were then calculated over all 10 runs. To foster our results, another validation was performed for the SVM, the DT, and the FNN. We conducted a leave-one-out approach on the user level, for all of the 518 included users, combined with a majority vote for the EMA-D questionnaires from the user that was left out, to see whether the prediction differs if the EMA-D questionnaires from the user who was left out are excluded. In conclusion, there was no obvious difference observed.

For all analyses, the following technical environment was used: a laptop with an i7 core (2.60 GHz); MATLAB, version R2017a (MathWorks); the Statistics and Machine Learning Toolbox (MathWorks); and scikit-learn, open source machine learning library, for Python (Python Software Foundation). For all applied methods, we used the default parameters of the technical environment. In MATLAB, the FNN, the SVM, and the DT were calculated, while in Python scikit-learn, the RFC was calculated.

## Results

### Overview

The four applied machine learning approaches showed different results for the research question. In general, the prediction accuracies were unexpectedly high.

### Feedforward Neural Network

The FNN was the worst-performing candidate. Here, for 72.67% of the EMA-D questionnaires, the mobile OS could be correctly predicted. In the MATLAB toolbox that was used, the essential parameter for the calculation was *feedforwardnet(10)*.

### Decision Tree

The DT, in turn, performed as the third-best candidate. It was applied with a 10-fold cross-validation and it predicted the correct mobile OS for 76.36% of the EMA-D questionnaires. Importantly, the resulting DT has a depth of 379, showing that the prediction can be categorized into a high-dimensional calculation. In the MATLAB toolbox that was used, the essential parameter for the calculation was *fitctree(X,Y,'CrossVal','on')*.

### Random Forest Classifier

The RFC performed as the best candidate; the mobile OS could be predicted correctly for 78.94% of the EMA-D questionnaires. In the Python scikit-learn method that was used, the essential parameter for the calculation was as follows: *RandomForestClassifier(n_estimators=100, bootstrap=True, max_features='sqrt', random_state=42)*. In addition to the prediction results, Table 4 shows the importance of the eight EMA-D questions for the overall prediction result of 78.94%; here, we used the *model.feature_importances_* feature of Python scikit-learn. Importantly, question 7 and then question 2 are the most important questions for the prediction result of 78.94%.

**Table 4:** Importance of the eight dynamic ecological momentary assessment (EMA-D) questions for the random forest classifier prediction. Question 1 (Q1): Did you perceive the tinnitus right now? (yes or no); Q2: How loud is the tinnitus right now? (slider); Q3: How stressful is the tinnitus right now? (slider); Q4: How is your mood right now? (manikins); Q5: How is your arousal right now? (manikins); Q6: Do you feel stressed right now? (slider); Q7: How much did you concentrate on the things you are doing right now? (slider); and Q8: Do you feel irritable right now? (yes or no).

Question Number	Q1	Q8	Q5	Q4	Q3	Q6	Q2	Q7
Percentage of Importance	0.03043	0.03985	0.08728	0.0913	0.17246	0.17425	0.19247	0.21194

### Support Vector Machine

The SVM performed as the second-best candidate. Overall, using all eight questions, the mobile OS could be predicted correctly for 78.65% of the EMA-D questionnaires. For the SVM, detailed results for single questions and question combinations are discussed in more detail. This will show that all eight questions are needed to get a prediction result with an accuracy that shows that the OS might be a confounder that should be further considered. The same detailed discussion could be accomplished for the other approaches, such as the RFC. We opted for the SVM for a more detailed discussion and to compare the results to other approaches to see if they deviate significantly from each other. More specifically, prediction results for combinations of two questions as well as single questions are shown in Table 5. Seven results will be further discussed. The discussion will show that the accuracies vary among the eight EMA-D questions on one hand. One the other hand, it will show that despite the observed variances, the overall achieved accuracy is high for different questions and their combinations.

First, we consider question 7—How much did you concentrate on the things you are doing right now? (slider)—and question 8—Do you feel irritable right now? (yes or no). They performed as the two best single questions for the prediction. Each of them has an accuracy of 58.80%. This result is only partly confirmed by the RFC. For the RFC, question 7 is also very important, but question 8 is less important for the RFC.

Second, question 5—How is your arousal right now? (manikins)—performed with the third-best result for a single question; here, an accuracy of 57.14% was attained. This question is like question 8, in that it is less important in the case of the RFC.

Third, the combination of question 7—How much did you concentrate on the things you are doing right now? (slider)—and question 8—Do you feel irritable right now? (yes or no)—performed as the best candidate for two-question combinations; in this case, an accuracy of 63.95% was achieved. This result is again only partly supported by the RFC (ie, for the RFC, question 8 was less important; see Table 4).

Fourth, the worst result was achieved when only using question 4—How is your mood right now? (manikins)—as the predictor. For question 4, an accuracy of 54.07% was achieved. Again, this deviates from the result of the RFC, where question 1 was the worst candidate.

Fifth, when solely combining *yes or no* questions (ie, question 1—Did you perceive the tinnitus right now?—and question 8—Do you feel irritable right now?), the mobile OS could be predicted correctly for 63.37% of the user assessments. This result also shows that without slider questions, a meaningful accuracy can be achieved.

Sixth, when looking at question-question combinations that include only sliders as answer types, the highest accuracies were achieved by the combination of question 2—How loud is the tinnitus right now? (slider)—and question 7—How much did you concentrate on the things you are doing right now? (slider). Here, an accuracy of 59.86% was achieved. This, in turn, is supported by the result of the RFC.

Seventh, it is remarkable that the overall prediction result with all eight questions is considerably higher than with single questions or combinations of two questions.

Finally, Table 6 represents the confusion table for the SVM calculations. Note that the values are for all eight EMA-D questions of the considered 14,708 Android questionnaires as well as 14,708 iOS EMA-D questionnaires.

**Table 5:** Prediction accuracies of the support vector machine (SVM) based on the eight dynamic ecological momentary assessment (EMA-D) questions and their combinations.

Question^a^ (Q)	Accuracy for each question (Q) combination, %							
	Q1	Q2	Q3	Q4	Q5	Q6	Q7	Q8
Q1	55.69	—	—	—	—	—	—	—
Q2	59.18	55.90	—	—	—	—	—	—
Q3	58.53	56.80	56.61	—	—	—	—	—
Q4	56.37	57.89	58.28	54.07	—	—	—	—
Q5	59.55	61.08	61.31	60.10	57.14	—	—	—
Q6	58.59	57.27	56.35	58.69	62.83	56.28	—	—
Q7	61.24	59.86	59.40	60.19	62.38	59.33	58.80	—
Q8	63.37	62.32	63.57	60.18	61.57	62.67	63.95	58.80

^a^Q1: Did you perceive the tinnitus right now? (yes or no); Q2: How loud is the tinnitus right now? (slider); Q3: How stressful is the tinnitus right now? (slider); Q4: How is your mood right now? (manikins); Q5: How is your arousal right now? (manikins); Q6: Do you feel stressed right now? (slider); Q7: How much did you concentrate on the things you are doing right now? (slider); and Q8: Do you feel irritable right now? (yes or no).

**Table 6:** Confusion table for the support vector machine (SVM) calculations over all eight dynamic ecological momentary assessment (EMA-D) questions.

Predicted class	Actual class			
	iOS		Android	
	True positives, n	False negatives, n	False positives, n	True negatives, n
iOS	13,002	N/A^a^	1967	N/A
Android	N/A	1706	N/A	12,741

^a^N/A: not applicable.

### Importance of Questions

**Figure 4:**
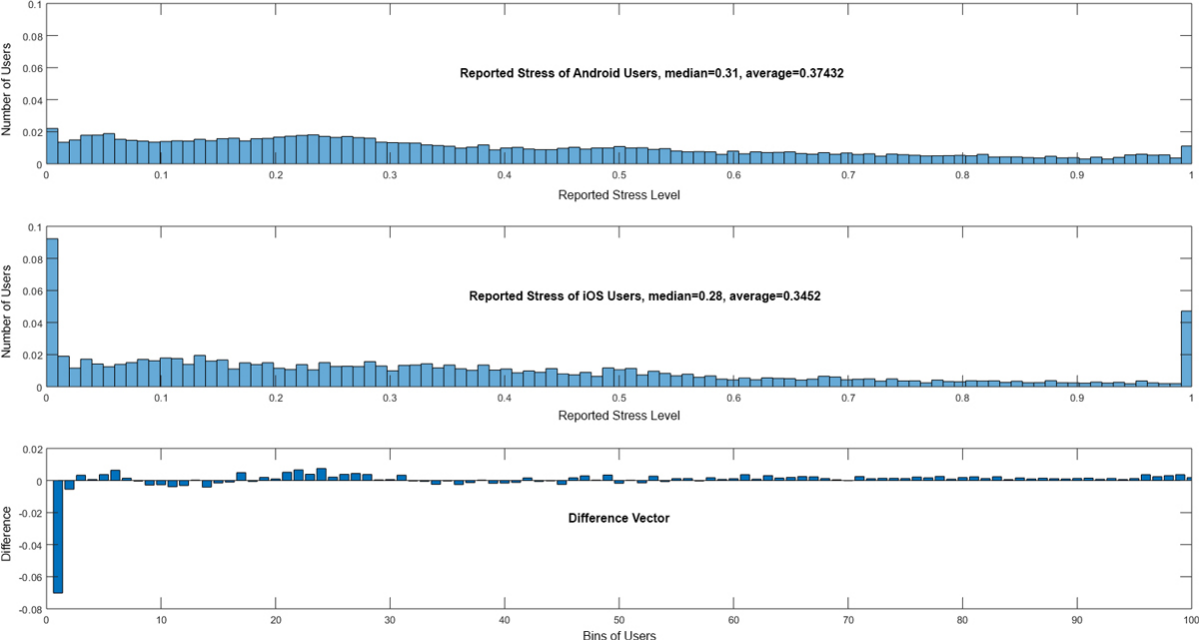
Answers to question 3—How stressful is the tinnitus right now? (slider)—and the difference vector of Android and iOS users.

**Figure 5:**
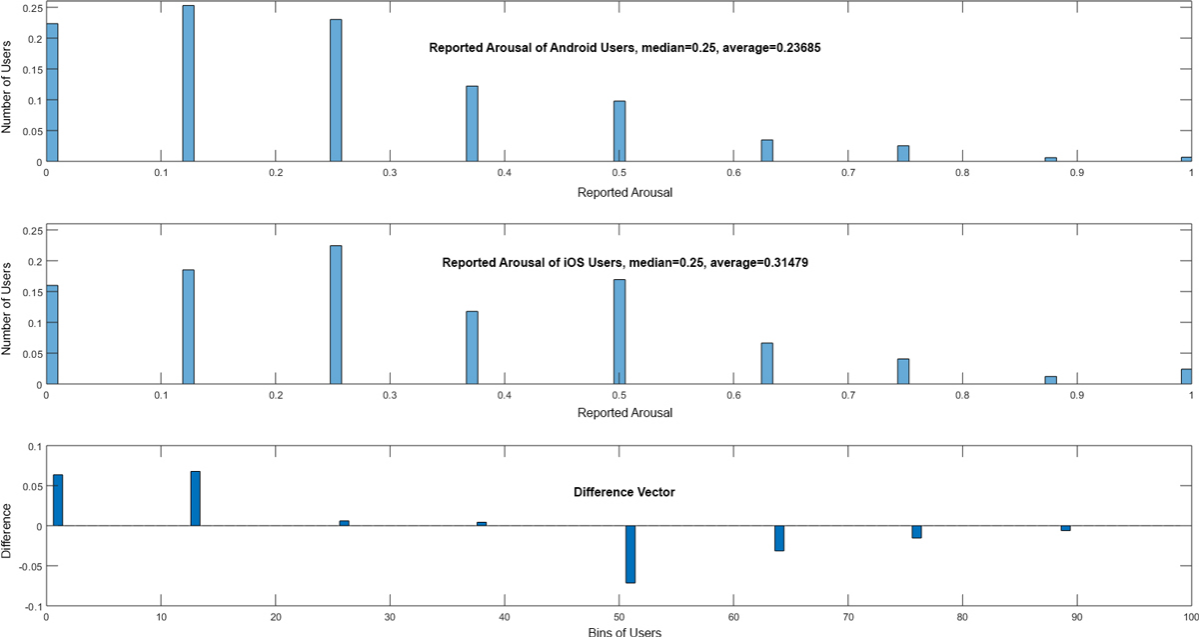
Answers to question 5 ”How is your arousal right now? (manikins)” and the difference vector of Android and iOS users.

In general, the question emerges as to why some of the eight EMA-D questions are better suited than others to correctly predict the mobile OS. One possible explanation refers to the answering behavior of the users of the two mobile OS types. To illustrate this, Figures 4 and 5 show, as examples, histograms of question 3—How stressful is the tinnitus right now? (slider)—and question 5—How is your arousal right now? (manikins). It is obvious that Android and iOS users answer differently.

**Figure 6:**
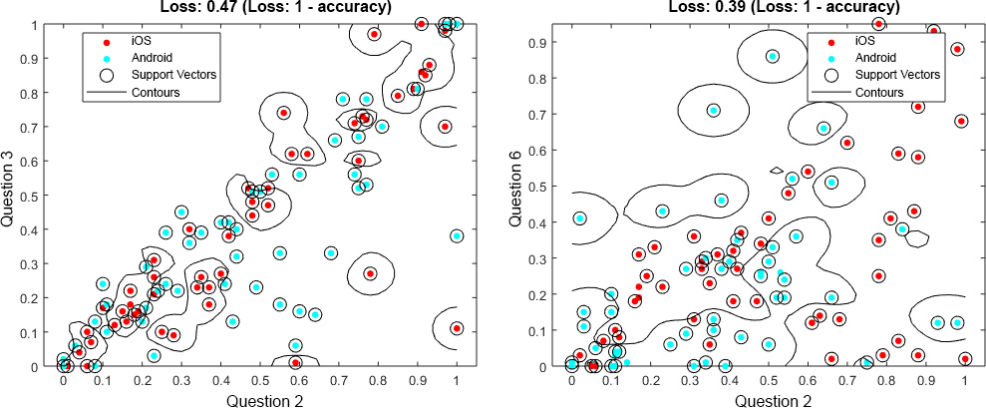
Support vector machine (SVM) results for question 2 (Q2), combined with question 3 (Q3) and question 6 (Q6), from 100 data entries. Q2: How loud is the tinnitus right now? (slider); Q3: How stressful is the tinnitus right now? (slider); and Q6: Do you feel stressed right now? (slider).

**Figure 7:**
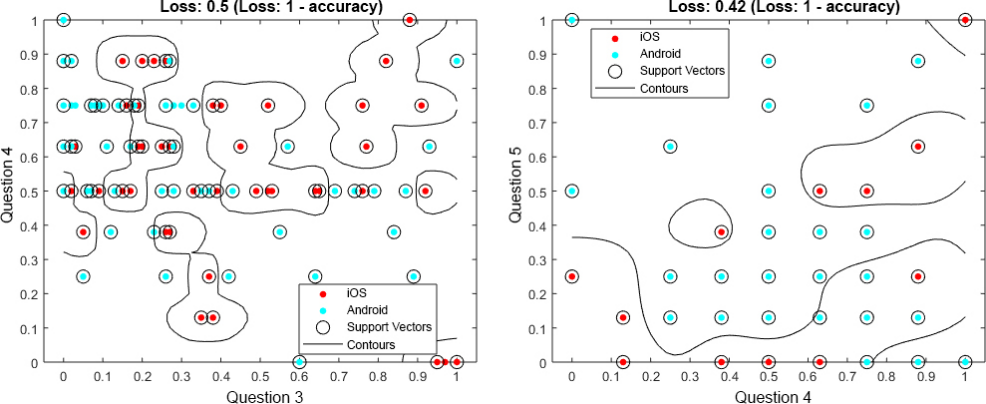
Support vector machine (SVM) results for question 4 (Q4), combined with question 3 (Q3) and question 5 (Q5), from 100 data entries. Q3: How stressful is the tinnitus right now? (slider); Q4: How is your mood right now? (manikins); and Q5: How is your arousal right now? (manikins).

Question 3 and question 5 have been chosen as examples, as they differ in their importance between the machine learning approaches: question 3 is the fourth-most important in SVM versus the third-most important in RFC, while question 5 is the third-most important in SVM versus the sixth-most important in RFC. In addition, other questions are more suitable for the overall prediction. Although they differ and other questions are better, they still show striking differences between assessments from Android and iOS users. To support this result, further consider Figures 6 and 7; they each show data for 100 users in total, distributed among Android and iOS. The data were randomly selected out of the entire dataset. This subset was chosen for the sake of clarity; if all data points were shown, less could be visually observed. In Figure 6, for question 2—How loud is the tinnitus right now? (slider)—in combination with question 3—How stressful is the tinnitus right now? (slider)—shown on the left-hand side of the figure, or question 6—Do you feel stressed right now? (slider)—shown on the right-hand side of the figure, the blue dots show the answers from the Android users, while the red dots show answers from the iOS users. It is striking that Android and iOS users answer differently. Furthermore, in Figure 7, for question 4—How is your mood right now? (manikins)—in combination with question 3—How stressful is the tinnitus right now? (slider)—shown on the left-hand side of the figure, or question 5—How is your arousal right now? (manikins)—shown on the right-hand side of the figure, the same can be observed. Importantly, Figures 6 and 7 are not representative of the entire dataset, but it is nevertheless notable that Android and iOS users answer differently. Further note that in Figures 6 and 7, we do not illustrate the achieved predictions. Instead, the attained loss is shown (ie, *1-loss* denotes the achieved accuracy). Furthermore, these combinations have been selected as they also show clear differences between Android and iOS assessments, although other questions have higher prediction accuracies.

## Discussion

### Principal Findings

This study evaluated whether it is possible to predict the mobile OS used by respondents for a provided EMA-D questionnaire based on the eight daily tinnitus questions included in the questionnaire, which was administered via TYT. Although the applied machine learning approaches showed different prediction results, in general, the achieved accuracies indicate that the mobile OS is a confounder that must be further considered. This confirms the investigated research question. We are able to predict the mobile OS used with high accuracy based on the dynamic daily assessment data. Compared to Pryss et al [12], the users’ ages were no longer different between Android and iOS users, which might be explained by the selection of the sample for this study: only users with more than 10 completed EMA-D questionnaires were selected. In addition to our prior works [12,14], this study shows that the mobile OS not only reveals insights into the tinnitus characteristics of the users, but it is possible to predict the mobile OS based on the provided daily TYT data. On top of this, widely used machine learning approaches with commonly used frameworks and without parameter tuning are able to predict the mobile OS with high accuracy. Note that the RFC achieved the highest prediction result of 78.94%, with default parameter settings using Python scikit-learn. In this context, question 7—How much did you concentrate on the things you are doing right now? (slider)—of the EMA-D questionnaire, which measures the concentration level of a TYT user at the moment, has especially revealed a high accuracy for the RFC prediction. In summary, four important results were found. First, the research question can be answered positively. We are able to predict the mobile OS used for a given EMA-D questionnaire with high accuracy using machine learning methods. Second, the prediction is possible with well-known machine learning methods and frameworks without parameter tuning. Third, machine learning indicates promising results on the EMA-D from TYT users. Therefore, this result should be exploited for further analyses. Fourth, when using mobile devices to collect clinically relevant data, the mobile OS used might be a confounder. Therefore, this information should be collected for each measurement and could be a relevant covariate in data analyses.

### Strengths and Limitations

In general, as a strength of this work, it could be shown that the technical peculiarities of different mobile OS types must be considered for the collection of clinically relevant data. As another positive aspect of this work, it could be shown that the types of answers for the questions do not necessarily indicate that a particular answer type, such as a slider, is used a priori with a bias. Otherwise, sliders or any other answer type would be more important than others. In general, we aimed at technically implementing TYT in a way that made sure the questionnaires looked identical on Android and iOS devices as well as having no default setting [11,28,29]. Despite this way of implementing the questionnaires visually, a potential bias cannot be excluded. Therefore, further investigations are required. For example, the sliders on Android and iOS have different numbers of decimal places. On Android, only 2 decimal places are stored, while on iOS, more than 2 decimal places are stored. For the investigation in this paper, the scales of all sliders were harmonized, but such differences must also be further investigated.

For the aspect of whether EMA-D can be used to predict not only the TYT assessments but the TYT users in general, we are conducting another study, in which we investigate whether we are able to predict the mobile OS used on the user level instead of on the assessment level. However, such investigation requires many more considerations. For example, how can we ensure that the training dataset users have similar characteristics as the users for which we apply the trained classifier? Note that such an investigation requires efforts regarding the frameworks used and their provided features.

### Conclusions

This work has shown opportunities on one hand and limitations on the other. A particular strength of this study is that TYT has a unique dataset, which is able to comprehensively compare Android and iOS OS types in a medical context. However, the different results between different machine learning approaches showed that it is difficult to predict which questions and answer types are, in general, appropriate for predictions. If a new platform shall be realized and one goal of the platform constitutes using machine learning methods for a prediction, this analysis has not revealed general guidelines that can be followed. Thus, these results can only be seen as a particular outcome for TYT. In addition, when gathering additional contextual information from the TYT users, such as geospatial data, new investigations become possible. In a recent work [27], for example, we investigated geospatial data of mobile crowdsensing users and whether their movement behavior could be a predictor for their current stress situation. As this work also revealed promising results, in the next version of TYT, GPS data can be gathered while filling out the EMA-D questionnaire, if a user allows this measurement.

In future work, we will further address the following three aspects. First, more studies must confirm the results of this work. Second, the results of TYT must be compared to other similar EMA datasets in order to confirm the results between different scenarios. Third, we need to conduct this study again based on the user level instead of on the assessment level.

However, if future work can confirm the presented results, then the combination of EMA, mobile crowdsensing, and machine learning seems to be a worthwhile research endeavor. Nevertheless, we are far from using the results of this work in clinical practice. On the other hand, together with already-revealed medical insights on TYT [6,37-40], the results of this work show that new opportunities are possible in the broader EMA and mobile crowdsensing contexts. In particular, EMA data that were gathered by mobile devices, as well as the crowdsensing paradigm, seem to be promising targets for the application of machine learning algorithms.

## Abbreviations

CSV: comma-separated values
DT: decision tree
EMA: ecological momentary assessment
EMA-D: dynamic ecological momentary assessment
EMA-S: static ecological momentary assessment
FNN: feedforward neural network
mHealth: mobile health
OS: operating system
RFC: random forest classifier
SF28: SmokeFree28
SVM: support vector machine
TYH: TrackYourHearing
TYT: TrackYourTinnitus
